# Supplementary material for: Impact of BMI on Survival Outcomes of Immunotherapy in Solid Tumors: A Systematic Review
Source: Int J Mol Sci. 2021 Mar 5;22(5):2628. doi: 10.3390/ijms22052628 (PMC7961496; doi:10.3390/ijms22052628)
Supplement: Supplementary file 1 [file ijms-22-02628-s001.pdf]

Supplementary Table S1. Records identified with the literature search through databases.

Database	Search strategy	Records Identified
	**MeSH terms/Search terms**	
PubMed	Body mass index<br>Obesity<br>Immunotherapy<br>Ipilimumab<br>Nivolumab<br>Pembrolizumab<br>Atezolizumab<br>Durvalumab<br>Avelumab<br>Melanoma<br>Carcinoma, non small-cell lung<br>Carcinoma, renal cell<br>Urologic neoplasms<br>Neoplasms	450
Scopus	Body mass index<br>Obesity<br>Immunotherapy<br>anti-CTLA4<br>anti-PD1<br>anti-PD-L1<br>Ipilimumab<br>Nivolumab<br>Pembrolizumab<br>Atezolizumab<br>Durvalumab<br>Avelumab<br>Melanoma<br>Carcinoma, non small-cell lung<br>Urothelial tumor<br>Carcinoma, renal cell<br>Cancer<br>Outcome	303

Web of Science	Body mass index<br>Obesity<br>Immunotherapy<br>anti-CTLA4<br>anti-PD1<br>anti-PD-L1<br>Ipilimumab<br>Nivolumab<br>Pembrolizumab<br>Atezolizumab<br>Durvalumab<br>Avelumab<br>Melanoma<br>Carcinoma, non small-cell lung<br>Urothelial tumor<br>Carcinoma, renal cell<br>Cancer<br>Outcome	150
Embase	Body mass index<br>Obesity<br>Immunotherapy<br>Ipilimumab<br>Nivolumab<br>Pembrolizumab<br>Atezolizumab<br>Durvalumab<br>Avelumab<br>Melanoma<br>Carcinoma, non small-cell lung<br>Carcinoma, renal cell<br>Urologic neoplasms<br>Neoplasms	252
Cochrane Central	Body mass index<br>Obesity<br>Immunotherapy<br>Ipilimumab<br>Nivolumab<br>Pembrolizumab<br>Atezolizumab	270

	Durvalumab<br>Avelumab<br>Melanoma<br>Carcinoma, non small-cell lung<br>Carcinoma, renal cell<br>Urologic neoplasms<br>Neoplasms	
